# The Right to Know and the Right Not to Know Revisited: Part One

**DOI:** 10.1007/s41649-017-0012-1

**Published:** 2017-07-13

**Authors:** Roger Brownsword, Jeff Wale

**Affiliations:** 1 King’s College London, London, UK; 2 Bournemouth University, Poole, UK

**Keywords:** Right to know, Right not to know, Genetic information, UK Biobank, Non-invasive prenatal testing

## Abstract

Prompted by developments in human genetics, a recurrent bioethical question concerns a person’s ‘right to know’ and ‘right not to know’ about genetic information held that is intrinsically related to or linked to them. In this paper, we will revisit the claimed rights in relation to two particular test cases. One concerns the rights of the 500,000 participants in UK Biobank (UKB) whose biosamples, already having been genotyped, will now be exome sequenced, and the other concerns the rights of pregnant women (and their children) who undergo non-invasive prenatal testing (NIPT)—a simple blood test that can reveal genetic information about both a foetus and its mother. This two-part paper is in four principal sections. First, we sketch the relevant features of our two test cases. Secondly, we consider the significance of recent legal jurisprudence in the UK and Singapore. Thirdly, we consider how, the jurisprudence apart, the claimed rights might be grounded. Fourthly, we consider the limits on the rights. We conclude with some short remarks about the kind of genetically aware society that we might want to be and how far there is still an opportunity meaningfully to debate the claimed rights.

## Introduction

Prompted by developments in human genetics, a recurrent bioethical question concerns a person’s ‘right to know’ and ‘right not to know’. For example, if A tests positive for Huntington’s Disease, do A’s close relatives (also A’s prospective employers or insurers) have the right to know about A’s test results?^1^ Conversely, does anyone have the right not to know?^2^ When genetic sequencing is increasingly affordable, when genetic information—thanks to big data sets and machine learning—promises to be increasingly interpretable, and when genetic information is readily available in a global marketplace,^3^ there are many stakeholders offering answers to these questions. In particular, there are patients and research participants (some wanting to know, others preferring not to know), clinicians and medical researchers (some wanting to disclose, others preferring not to), and various commercial interests and lobbyists (some supporting the rights, others opposing them).

In this paper, building on previous work (Wale 2015; Brownsword 2016; Brownsword and Wale 2016, 2017), we will revisit the claimed rights in relation to two particular test cases. The first concerns the rights of the 500,000 participants in UK Biobank (UKB) whose biosamples, already having been genotyped, will now be exome sequenced. The second, arising from the discovery that fragments of foetal DNA circulate in the mother’s blood, concerns the development of non-invasive prenatal testing (NIPT)—a simple blood test that can reveal genetic information about both the foetus and the baby’s pregnant mother. With the use of NIPT now being piloted within the UK national screening pathway for Down’s syndrome, there are pressing questions about the extent of the mother’s rights.

The paper is in four principal sections (and it is published in two parts). First, we sketch the relevant features of our two test cases. Secondly, we consider the significance, relative to the claimed rights, of the UK Supreme Court’s decision in *Montgomery v Lanarkshire Health Board* 4 and, concomitantly, of the Singapore Court of Appeal’s recent decision in *Hii Chii Kok v Ooi Peng Jin London Lucien* 5 where, subject to some minor revision, the approach in *Montgomery* is adopted. Thirdly, we consider how, the jurisprudence apart, the claimed rights might be grounded. Fourthly, we consider the limits on the rights (including some short thoughts concerning the constraints on regulatory effectiveness). We conclude with some short remarks about the kind of genetically aware society that we might want to be and how far there is still an opportunity meaningfully to debate the claimed rights.

However, we preface our discussion with a general caveat. In revisiting these claimed rights, we are conscious that we presuppose a culture in which the idea of individual rights is central to legal and bioethical discourse, a context in which the healthcare system is relatively well resourced, and a community in which chromosomal disorders are a high priority for the use of NIPT. Such conditions, we are well aware, are not universal. While we hope that our discussion will inform bioethical thinking, we recognise ‘the importance of local context when thinking about the global expansion of new genomic technologies.’(Mozersky et al. 2017).

## Two Test Cases

We have already introduced our two test cases, UKB (as a major research resource) and NIPT (as a genetic test that already has clinical applications). However, we need to say a bit more about them so that the context for our discussion is clear.

### UK Biobank

Briefly, UKB curates biosamples and data derived from its 500,000 UK participants. Bona fide researchers worldwide may, and do, access the resource for health research purposes. Initially, there was no plan for *systematic* sequencing or genotyping of the samples; rather, the hope and the expectation were that the genetic data held by the resource would be generated by particular researchers who would use the samples for their own projects and then return the sequenced or genotyped data to the resource. However, in recent years, there have been two significant initiatives to develop the resource in a more systematic way. First, additional funds were made available for the genotyping of all participants’ samples, targeting some 800,000 markers and enabling the imputation of millions of single nucleotide polymorphisms (SNPs). In June 2015, UKB released the data on 150,000 of its participants, with the data on the remaining 350,000 participants due to be released in 2017. Secondly, there is to be a major investment by GSK and Regeneron in exome sequencing 50,000 UKB participants as the first stage in sequencing all 500,000 over the next 5 years.^6^

In practice, because individual participants can be linked by UKB to their data and samples, they could be given access to their genetic information—for example, a participant, worried about possible loss of cognitive function, might want to know whether he has the ApoE4 marker for Alzheimer’s disease^7^, and if clinically significant findings are made by researchers, they could be returned to the particular participant. However, the terms and conditions for participation in UKB provide that there will be no feedback of research findings to individual participants. Accordingly, if we assume that, prior to participation, individuals have the right to know, then the deal is that one waives the benefit of that right.

At the same time that UKB has been undertaking the genetic development of the resource, it has also begun a ground-breaking multi-modal imaging project to which it plans to recruit 100,000 of its participants. Here, the protocol provides for the return of potentially serious clinical findings (where they are noticed by the radiographers who take the images and then confirmed, on referral, by radiologists) to participants with no option to opt out. Again, if we assume that, prior to participation in the imaging study, individuals have the right not to know, then the deal is that one waives the benefit of that right.

If we juxtapose the implicit waiver of the benefit of the right to know (in the main study) with that of the right not to know (in the imaging study), then this invites an explanation. Granted, the exercise of one of the rights necessarily disapplies the other, but why is it that, where a radiographer happens to notice a potentially serious pathology (confirmed by a radiologist), participants are told but not so if a researcher happens to notice a similar thing when working with the biobank images, data or samples? The crucial question, however, is whether, if the claimed rights are recognised, the participants’ waivers will survive ethical and legal review. This, we take it, will turn on whether the consent given by participants is sufficiently free and informed or whether UKB has taken all reasonable steps to ensure that participants understand the protocol.^8^

### NIPT

As we have said, NIPT is being piloted within the UK national screening pathway for Down’s syndrome.^9^ The attraction of the test is that it promises to reduce the need for an invasive amniocentesis test or chorionic villus sampling and, with that, to reduce the number of babies lost during pregnancies (Chitty 2016). However, because NIPT presents an opportunity to provide information about the foetus that goes beyond the trisomies,^10^ even to the point of full genomic profiling, as well as returning information about the mother,^11^ it invites questions about how broad the test should be and how far the mother’s right to know might extend. For example, should NIPT be used to provide information about relatively trivial conditions as well as non-health related information (such as eye/hair colour or gender) about the developing child?

In the case of the trisomies, NIPT can be very reliable, although subsequent tests may be required to achieve a truly diagnostic outcome, but in other cases—such as some chromosomal microdeletions (missing genetic information) and microduplications (additional duplicated genetic information)—the results may be equivocal, hard to interpret and fall a long way short of diagnostic criteria. Nevertheless, parents can already commission these tests through the private sector and seek a range of information.^12^ Meanwhile, the public health sector often plays catch up, due partly perhaps to a reluctance to embrace the ‘full menu’ offered privately. Such reluctance might be explained by an unwillingness to fund tests yielding uncertain information or choices that present unlawful and difficult options for parents. States may also prefer to operate a contingent model where publicly funded testing is only offered as an option for certain risk groups.^13^

Predictably, the availability of NIPT exacerbates concerns about the ‘medicalisation’ of pregnancy, the ‘commodification’ of life, the ‘trivialisation’ of decisions about abortion, the ‘routinisation’ of prenatal testing, the ‘stigmatisation of disability’ and so on.^14^ Responding to such concerns, the Nuffield Council on Bioethics has recently reported on the ethical issues raised by potential future uses of NIPT.^15^ Although the Council does not explicitly frame the bioethical issues in terms of whether and what a pregnant woman has the right to know, or not to know—rather, framing the questions in terms of accommodating the values of choice, autonomy and consent, the avoidance of harm and equality, fairness and inclusion^16^—questions about the claimed rights are inevitably provoked by its discussion.

## The Recent Jurisprudence

In common law legal systems, a patient or a participant who believes that they have been wronged by the withholding or disclosing of genetic information might seek a foothold in the general jurisprudence. Thus far, at any rate in English law, the question of whether one might succeed in a tort claim were screeners who use NIPT to decline to return findings that one wished to have (or if they were to return findings contrary to one’s known wish not to have feedback) has barely surfaced. By contrast, there has been much discussion of whether a participant might succeed against a biobank researcher in a tort claim for wrongful non-disclosure of relevant individual findings.^17^ The consensus is that English law—in which the key legal question is whether it would be ‘fair, just, and reasonable’ to place researchers under a feedback responsibility—does not clearly support such a claim.^18^ However, following the important decision of the UK Supreme Court in *Montgomery v Lanarkshire Health Board,* 19 it is arguable that, in the clinical context of NIPT if not in the research context of UKB, there is new support for the right to know. Moreover, given that the Singaporean Court of Appeal has very recently followed the approach in *Montgomery*, it is arguable that, in Singapore too, there is new support in the jurisprudence for the right to know.

The principal question in *Montgomery* was whether a pregnant woman, who was a diabetic, should have been informed that there was a risk of shoulder dystocia and given the option of delivery by caesarean section. Instead, as the court narrated the story,^20^ she was not made aware of this particular risk, and sadly, the risk eventuated during an attempted vaginal delivery resulting in the baby being born with severe disabilities. Differing from the lower courts, where a traditional physician-deferential *Bolam/Bolitho* 21 test had been applied, the UK Supreme Court held that the relationship between clinicians and patients must be rights respecting rather than paternalistic and that patients have the right to be informed about their options (together with their relative benefits and risks). Signalling a distinct movement away from medical paternalism and patient dependence, the new approach is built on mutual rights and responsibilities, treating patients ‘so far as possible as adults who are capable of understanding that medical treatment is uncertain of success and may involve risks, accepting responsibility for the taking of risks affecting their own lives, and living with the consequences of their choices’.^22^ That said, *Montgomery* recognises that, in exceptional circumstances, doctors may legitimately withhold information under cover of the so-called ‘therapeutic privilege’. However, the Court emphasises that this exception ‘is not intended to subvert [the general principle] by enabling the doctor to prevent the patient from making an informed choice where she is liable to make a choice which the doctor considers to be contrary to her best interests’.^23^ In short, patients have the right to make their own judgments, prudential and moral, of what is in their best interests,^24^ and it is the responsibility of doctors not to override these judgments but to assist patients by ensuring that their choices are suitably informed.

As with any potentially landmark decision in the law of negligence, lawyers can read the ratio (the governing principle) of *Montgomery* in more than one way. Those who wish to minimise the impact of the decision will read it narrowly, while those who wish to build on it will read it more broadly.^25^ Nevertheless, after *Montgomery*, we suggest that it is reasonable to assume that, at all stages of a pregnancy, a woman has the right to be informed about her options. It follows that, once NIPT is embedded in the screening pathway, pregnant women will have the right to know about the availability of the test and to be informed about its risks and consequences. The fact that *Montgomery* supports the right to know in relation to the primary results is probably not especially significant because they will be returned anyway. The real question is whether *Montgomery* supports a more extended application of the right to know.

Suppose, for example, an NIPT screen reveals a potentially life-threatening condition that affects the *mother*. While *Montgomery* does not directly support the woman’s right to be informed, it certainly does not weigh against it and, arguably, by analogy with the case of an easy rescue, she is entitled to be informed.^26^ Similarly, *Montgomery* does not directly support the woman’s right to be informed if NIPT reveals information about the foetus other than that relating to the trisomies. Nevertheless, with the courts realising the importance of the common law being in line with fundamental values, a court might in future start with the proposition that, if a woman wishes to access information about the genetic profile of her baby, she has the right to do so.

As for the right not to know—which, of course, will only become an issue where the practice is to return findings—the thinking in *Montgomery* suggests that, on the one side, healthcare professionals must restrain any paternalistic impulses that they might have, and on the other, patients must live with the consequences of their decisions. However, it might not be quite so easy to turn back the tide of genetic information. For example, the costs and inconvenience of administering the right not to know might not be trivial, professionals might find it difficult to accept that they should act as though patients know best when in their expert judgement they manifestly do not and the reproductive culture of communities in the future might be far more risk-averse.^27^

Before we attempt to take stock, we need to consider the Singapore Court of Appeal’s recent decision in *Hii Chii Kok v Ooi Peng Jin London Lucien* 28 where, broadly speaking, *Montgomery* is adopted.^29^ In *Hii Chii Kok*, the Court recognised that, while there is a dynamic relationship between patients and clinicians, the interactions between the parties basically relate to diagnosis, advice and treatment, and indeed, the claimant in the case alleged that the defendants had been negligent in each of these departments—that is, in their diagnosis of his condition, in their advice to him and in their treatment of him. Applying the traditional *Bolam/Bolitho* test to the matters of diagnosis and treatment, the Court followed the trial judge in dismissing these claims. However, the Court took the patient-centric approach in *Montgomery* to be the appropriate standard in relation to the matter of advice. While this did not actually avail the claimant—who, as found on the facts, was fully informed and properly advised, whether judged by a responsible body of medical opinion (as per the *Bolam/Bolitho* test) or by the more patient-sensitive *Montgomery* test—this is clearly an important development in the local jurisprudence.

In a perceptive passage, the Court in *Hii Chii Kok* relates the shift from *Bolam/Bolitho* to *Montgomery*, to a new prioritisation amongst the key bioethical principles famously adumbrated by Tom Beauchamp and James Childress.^30^ Instead of prioritising (paternalistic) medical practices designed, in line with the principle of beneficence, to improve the health and well-being of patients, the priority in advising patients is to respect their autonomy. The point, as the Court strikingly expresses it, is not ‘whether there is a duty to ensure that information is shared with the patient’^31^—that there is such a duty is not in doubt. However, the question is whether the information to be shared is to be determined by prevailing medical practice and opinion or by reference to the patient’s perspective concerning what is material. Answering that question, the Court first says that ‘once we accept that a patient should be equipped with such information as is reasonably required to arrive at an informed decision, it would be incongruous to then *ignore* the patient’s perspective when examining the question of the sufficiency of the information provided,’^32^ which then leads to the proposition that ‘the patient has a prima facie right to the information reasonably required to enable him to make a decision.’^33^ In other words, if the doctor has, but withholds, information that is reasonably required by the patient, then that is a prima facie infringement of the patient’s right to know and the burden of justification is on the doctor.

In what circumstances might a doctor justifiably withhold relevant information from a patient? The Court mentions the following three non-exhaustive possibilities^34^: (i) where the patient waives the benefit of the right (i.e. consents to the information being withheld), (ii) where a medical intervention is justified under the principle of necessity—such as where life-saving surgery is performed on a person who temporarily lacks decision-making capacity and (iii) where the therapeutic privilege applies. As in *Montgomery*, the Court cautions against abuses of the therapeutic privilege.^35^ For our purposes, though, it is the Court’s short remarks on the first possibility that are of most interest. Here, the Court says:

Patient autonomy confers rights on the patient; it does not impose obligations. Thus, there is no *obligation* on the patient to hear what is material to him, and he is entitled to exercise his autonomy by deciding that he does not wish to hear further information about the proposed treatment or its alternative. Given the seriousness of such a decision, waiver should ordinarily be express, or *extremely* clear if it is to be inferred. Moreover, the doctor should satisfy himself that, in deciding to waive his right to hear further information, the patient properly appreciates the seriousness of his decision.^36^

Hence, although the Court’s underlying bioethical reference is the principlism of Beauchamp and Childress, its privileging of autonomy leads to the crystallisation of a patient’s right to know, and as with any will or choice-based understanding of rights, the patient rights holder is entitled to waive the benefit of the right. While this does *not* entail recognition of a patient’s right not to know, it certainly puts the patient’s autonomy interest and his right to know front and centre.

Taking stock: first, in relation to the woman’s right to know the primary results of NIPT, both *Montgomery* and *Hii Chii Kok* are clearly supportive, but it remains to be seen how far this recognised right might extend beyond these results (which, anyway, will routinely be returned). Secondly, it is unclear that these decisions support the woman’s right *not* to know. Thirdly, although *Hii Chii Kok* underlines the need for unequivocal signalling of a waiver, we do not know how demanding the courts will be in applying the general bioethical principle that a waiver, or a consent, is valid only where the rights holder gives it ‘freely’ and on an ‘informed’ basis.^37^ Moreover, neither *Montgomery* nor *Hii Chii Kok* can give us any assistance in knowing how courts will apply their local legislative controls on unfair contract terms or their case-law on unconscionable terms or volenti non fit injuria. No doubt, there are many nice points of law that might arise here. Fourthly, it remains moot whether these precedents should, or will, have any traction in *research* contexts such as that of UKB. Accordingly, while, in their respective jurisdictions, *Montgomery* and *Hii Chii Kok* might offer a necessary foothold in the jurisprudence, beyond the clear case, we need further reasons to clinch the argument for the claimed rights.^38^

## The Basis of the Rights

On what basis, other than in case-law developments of the kind that we discussed in Part One of the paper, might one claim, or indeed contest, the right to know or the right not to know? In some contexts, the claimed rights might have a basis in either hard or soft law—for example, in particular pieces of informational legislation (dealing with, say, freedom of information or a data subject’s rights in data protection law), or in provisions such as Article 10.2 of the Convention on Human Rights and Biomedicine, according to which: ‘Everyone is entitled to know any information collected about his or her health. However, the wishes of individuals not to be so informed shall be observed’.^39^ Other things being equal, there would also be a reasonable transactional basis for the claimed rights if one had been promised that one would be told or would not be told some particular matter—as where non-invasive prenatal testing (NIPT) is procured privately or as in the terms and conditions for the Estonian Genome Project^40^ which provide explicitly and exceptionally that participants have both claimed rights.^41^

With regard to our two test cases, there are of course many differences between the clinical and reproductive context in which NIPT is being piloted and the multi-purpose health research context of UK Biobank (UKB). Whereas, in the former, it is clear that pregnant women will be given the results of the test—because, without knowing that they have a negative result, they cannot be confident in declining a more invasive and risky test—in the latter, the extent to which the claimed rights apply seems to hinge on the particular terms and conditions for participation (and the adequacy of participants’ consent or waiver).

Where the position is not clear, how might the claimed rights be grounded? In what follows, we sketch two arguable grounds: first, on the basis that one has a reasonable expectation that one’s interest in knowing or not knowing about one’s genetic profile should be protected and privileged, and secondly, that the claimed rights are already immanent in principles or concepts that we recognise.

### The Claimant Has a ‘Reasonable Expectation’ That the Information at Issue Should Be Disclosed (a Right to Know) or Not Disclosed (a Right Not to Know)

Claiming the rights on the basis that one simply has the relevant expectation is not sufficient; that expectation must also be a reasonable one. How might one’s expectation be shown to be reasonable?

First, it might be claimed that the researchers or screeners had informally signalled that feedback would be given. In relation to NIPT, where the anticipated benefits of the test depend upon the results being returned, the context clearly raises a reasonable expectation that the primary results will be disclosed. By contrast, many biobanks, including UKB, have well-advertised ‘no feedback’ policies, suggesting that participants have no reasonable expectation of individual findings being returned to them. Even though such policies might be expressed ambiguously in terms that leave it unclear whether the biobank regards itself as having a duty *not* to return findings or as having *no duty* to return findings (thereby retaining a discretion to return findings), it is clear that ‘no feedback’ amounts to a denial of participants having a claim-right to know.

Secondly, in the context of biobanking, a participant might plead an expectation, shared by others, that because participants assist researchers in various ways, the latter should reciprocate by giving appropriate feedback.^42^ However, the fact that others share A’s expectation does not make anyone’s expectation *reasonable*. The underlying question is whether there is a generally accepted principle of reciprocation and whether, relative to such a principle, it is reasonable to expect feedback to be given.

Thirdly, settled custom and practice might be relied upon, whether with regard to the use of NIPT or in biobanking. Even if the screeners or researchers with whom one deals have not signalled their position in relation to the return of findings, the sectoral custom and practice (of biobanking and screening) might suffice to ground a reasonable expectation that the claimed rights are recognised. Or, echoing the previous point, one might argue that, beyond sectoral practice, it is customary to respect the principle of reciprocity, of one good turn deserving another—a principle that would surely aid claims made by participants in UKB who contribute enormously to the project but not so obviously claims made by patients who have given blood for NIPT purposes.

### The Claimed Rights Are Immanent Within Recognised Concepts or Principles

Irrespective of such contingent indicators, might the claimed rights be immanent within such key concepts as property, privacy, autonomy and agency (and our understanding of their associated rights)?^43^

Arguably, both claimed rights can be teased out of an agent’s right to self-development. This implies the right to have conditions that allow for the free construction of one’s personality (or identity)—that is to say, each agent being free to be who they want to be, to form the relationships that they want to have, and to pursue the interests that they choose to have and so on.^44^ Such a critical interest includes an interest in informational self-determination that bridges both well-being and autonomy. In an age of burgeoning genetic information, some agents, preferring to be aware of risks and to manage them, will want to know as much as they can about the details of their genetic profile, but others will prefer not to anticipate their futures and to cross whatever bridges that have to be crossed as and when they meet them—23andMe will not be for them. While the former will claim the right to know, the latter will insist on the right not to know. However, both rights are grounded in the root interests of human agents, and if denied, there is a diminution in both agent autonomy and agent well-being.^45^

This kind of argument is nicely illustrated in a recent exchange concerning a child’s right to know their genetic origins (particularly that they were donor-conceived). According to Ravitsky, Guichon, Lemoine and Giroux, the question is not whether, in a consequentialist way, children would be better off knowing, or not knowing, such things; rather, there is a question of autonomy at stake (Ravitsky et al. 2017). Thus,

Violating [autonomy] deprives donor-conceived people of the liberty to choose what meaning they assign to the genetic components of their identity and relationships, a choice experienced and taken for granted by most others in society. The deontological framework does not claim that all or most donor-conceived people will necessarily find their genetic origins of great importance, but rather that they are entitled to make that determination for themselves.^46^

Indeed, if we are looking for a knock-down argument, as Ravitsky et al. argue, if we recognise the right to know in adoption law, then why not also in assisted conception law? And, if in both adoption and assisted conception, then why not in non-adoptive, non-assisted, conception, and so on?

Alternatively, we might try to anchor the claimed rights to our thinking about property and about privacy. In both cases, we might argue that recognition of the claimed rights coheres with our understanding of these concepts. For example, we might argue that the right to know is already immanent in our thinking about property. Here, the idea is that, if anyone has the right to know the results of genetic analysis of a sample, it is the person who gave the sample^47^—or, to put this somewhat tendentiously, but perfectly naturally, it is the person whose sample this is. To be sure, the introduction of proprietary rights in this way is heavily contested. Nevertheless, if an agent can simply say in relation to such information: ‘This sample (and the information it contains) is my property; it is mine’, this readily explains and justifies the demand to have findings returned. Or, we might argue for the right not to know by relating it to the protean concept of privacy. On almost any version of privacy—such as the right to maintain a state of psychological separateness, or the right not to be subject to unwilled ‘intrusions’, or the right to be let alone—it seems plausible to argue that we each have the right not to know about our own genetic profile.^48^

## Specifying the Limits of the Rights

If insurers argue that they have the right to know about the genetic profiles of their prospective insured, they can draw on the general legal principle that requires full disclosure by applicants. However, even where the right to know is recognised, it might be limited (or outweighed) by conflicting rights (such as the right against unfair discrimination). Indeed, one reading of *ABC v St George’s Healthcare NHS Trust & Ors* 49 is precisely whether the duty to warn the patient (correlatively, the patient’s right to know) was limited (or outweighed) by the duty to respect the fact that the relevant information was obtained in confidence. Our next question is this: What limits might be specified *within* the claimed rights themselves?

Although our question relates to both claimed rights, we restrict our discussion to the right to know. We consider three possible limits on the scope of this right, namely: (i) the information might be applied for an unlawful purpose, (ii) the condition to which the information relates is not sufficiently serious to warrant disclosure and (iii) the claimant might be harmed because the information given is not sufficiently interpretable to be actionable.

### Unlawful Purposes

It is unlikely that personal health findings returned by a biobank would be applied for unlawful purposes. However, the results of NIPT might be acted on unlawfully, most obviously by terminating the pregnancy for social sex selection reasons.^50^ Recognising this particular risk, the Nuffield Council on Bioethics has recently recommended that NIPT providers should be prohibited from generating or reporting information about the sex of the foetus unless ‘there is concern that the foetus may be showing signs of a significant sex chromosome aneuploidy or is at risk of a sex-linked disorder.’^51^

However, the Council’s view notwithstanding, there surely will be cases where providers—particularly private extra-jurisdiction providers—will return results that identify the sex of the foetus. Moreover, such providers can argue, first, that the local certifying doctors rather than remote NIPT providers are the appropriate gatekeepers of lawful terminations, and secondly, that many women simply want to know (but not act on knowing) the sex of their baby.^52^ Of course, if providers know full well that a woman will use her NIPT results for unlawful purposes, the second of these rejoinders will be hollow, but then providers who do not want to have such knowledge will no doubt refrain from asking too many questions.

Where NIPT providers are operating in a global online marketplace for genetic information, we have to lower our expectations about the effectiveness of regulatory interventions. However, this is not entirely a counsel of despair. For example, internet service providers, or other online intermediaries, might be prepared to act as ‘chokepoints’, restricting supply of goods, services or information from target sites; (Tusikov 2016) local regulators might be able to target relevant assets or personnel in the home jurisdiction; and, there might be opportunities for reciprocal cross-border enforcement or other forms of cooperation between national regulators.^53^

### Non-serious Conditions

We noted earlier that UKB is giving some feedback to those who participate in the imaging study. The feedback is limited to conditions that are potentially serious. To some extent, this might be seen as paternalistic, judging that as a general rule the distress occasioned to participants by false alarms will outweigh the benefits. However, because participants who are alerted are advised to follow this up with their doctors, there is also a non-paternalistic cost consideration: Quite simply, the NHS does not have the resources for UKB to be encouraging participants to add unnecessarily to the strain on the system.

Similarly, the Nuffield Council on Bioethics has recently recommended that NIPT should not normally be used to test ‘whether a foetus has a less significant medical condition or impairment or an adult onset condition; to find out whether the foetus is the carrier of a gene for any kind of medical condition or impairment; nor to reveal non-medical traits of the foetus.’^54^ Such restrictions might be read as hinting at moral paternalism—that is to say, at the Council judging that it is not in a woman’s moral interest to have this kind of information which might then be used as an inadequate reason for terminating a pregnancy. However, the limitation might also be justified in much the same (resource-related) way that UKB might justify its limited feedback to participants. Moreover, in the typical case, where the information concerns the foetus, the benefit/harm calculation which is implicit in the Council’s reasoning is elastic enough to include undermining the capability of the future (as yet unborn and quite possibly to be terminated) person to make their own choices (including not to know) and any negative impacts on the ‘wider societal environment’ (whatever this might mean).^55^ In other words, the Council might defend its apparently paternalistic recommendations by representing the case for limitation in non-paternalistic terms; rather than limiting the range of NIPT for the sake of the woman’s own moral welfare, the purpose is to protect against the risk of harm to the foetus-that-becomes-a-person and the social environment. Nevertheless, quite why the Council—which, after all, was amongst the first to draw attention to the right to know^56^—should shy away from a more direct and explicit engagement with the claimed rights in favour of an apparently more paternalistic and certainly more consequentialist approach is a matter for further consideration (but not in this paper).^57^

Again, the fact that private online providers are in play presents regulatory challenges. Ideally, post-test, there should be a regulatory framework that (i) makes provision for the storage, security and future destruction of important personal data (including genetic data) and (ii) treating both the present mother and any future child as potential claimants, takes an acceptable position on their respective right to know and not to know.

With regard to the former, there are obvious advantages in a coordinated global approach that encourages (if not requires through licence or other means) providers to be transparent about their processes for handling/storing data and the mechanisms for addressing any future areas of dispute. With regard to the latter, while domestic regulators can consult with and then be guided by their community’s view of reasonable informational expectations, the positions taken are likely to differ from one state to another. In some places, the right of a present generation mother to know might be treated as taking priority over the rights of future generations to choose whether or not to know, but in other places, this priority might be reversed. In the face of such difficult bioethical choices, coupled with significant local variation, global agreement might be simply wishful thinking.

### Interpretability and Actionability

The Nuffield Council on Bioethics recommends limiting use of NIPT to cases where it provides an ‘accurate prediction’ of a serious medical condition or impairment.^58^ Similarly, it is common amongst biobankers to find a reluctance to return genetic data where it is difficult to interpret.

Recognising the potential uncertainty of genetic information, the different degrees of seriousness of findings, and different grades of actionability,^59^ rather than opting for no feedback or full feedback, one suggestion is that we might place findings on one of three lists: (1) a ‘white list’ (results should be returned), (2) a ‘grey list’ (results may be returned) and (3) a ‘black list’ (results should not be returned).^60^ As researchers improve their understanding of the significance of particular genetic markers, the lists would be revised with some markers moving onto the white list. In this way, the need for paternalistic restriction would be reduced.

Certainly, where the test is publicly funded, a policy of returning findings that are not properly understood would seem like poor value for money. However, if individuals wish to use their own funds to procure such information, and if (like the Nuffield Council on Bioethics) we commit to the value of ‘choice, autonomy and consent’, while the State might not feel justified in prohibiting such action, it might at least see good reason not to encourage it—and, especially so, if reliance on privately procured tests leads to unintended burdens on public resources.

## Conclusion

In a democracy, as Wendell Wallach insists, citizens reasonably expect to have the opportunity to give their approval to the technological futures that are being created.^61^ To do this, ‘an informed conversation must take place’^62^, but as Wallach also notes, the windows of opportunity for holding such a conversation can be narrow.

In some places, the demand from patients and research participants for the return of findings, coupled with commercial pressure to supply and to have access to such information as well as an increasing professional reliance on genetics might be such that, in relation to the right to know, it is already too late to debate the kind of society that we want to be—the fact of the matter is that the direction of travel is already strongly towards a society that is genetically literate, informed and curious, with the return of genetic results as the norm.

By contrast, so far as the right not to know is concerned, all might not be lost. Privacy, albeit culturally contingent, is not yet dead, and there might still be time to have an informed conversation about this right. Where this is so, then we should heed Wallach: The window for this conversation is now. If citizens are to have more than a straight choice between living off-grid like the Amish or participating fully in the geno-world, then bioethicists need urgently to debate the still elusive right not to know.

## Footnotes

1 On which, see *ABC v St George’s Healthcare NHS Trust & Ors* [2015] EWHC 1394 (QB), [2017] EWCA Civ 336. In this case, the claimant, who was pregnant at the relevant time, sued the defendants, complaining that they had failed to inform her that her father had been diagnosed with Huntington’s Disease. Had the claimant been so informed, she would have known that she was at risk of having the disease, and knowing that her children would also be at risk, she would have terminated the pregnancy. In the High Court, the claim was struck out (as unarguable) on the ground that, because the defendants obtained the information about the father’s health status in confidence, and because the father was emphatic that he did not want his daughter to be told, it would not be fair, just, and reasonable to impose on them a duty to inform the daughter. For comment, see (Chico 2016; Fay 2016). Not altogether surprisingly, the Court of Appeal has now reversed this decision and remitted the case for trial.

2 See, e.g. the Nuffield Council on Bioethics 1993 and Chadwick et al. 2014.

3 Kolata 2017: see https://www.nytimes.com/2017/04/06/health/fda-genetic-tests-23andme.html?_r=0 (last accessed April 7, 2017).

4 [2015] UKSC 11.

5 [2017] SGCA 38.

6 See: http://uk.reuters.com/article/uk-health-genes-gsk-regeneron-pharms-idUKKBN16U01O (last accessed March 28, 2017).

7 See: https://en.wikipedia.org/wiki/Apolipoprotein_E (last accessed June 15, 2017).

8 Given that the general rule is ‘no feedback’, the standard for reasonable notice might be quite demanding. See also Laurie et al. (2010).

9 See: Gallagher 2016.

10 For example, Sequenom’s MaterniT 21 PLUS 2017.

11 See, e.g. Oswald 2015.

12 Such as Sequenom’s MaterniT 21 PLUS test (see: Sequenom’s MaterniT 21 PLUS 2017).

13 The current UK pilot offers NIPT following conventional screening tests (including serum screening and ultrasound) to high risk groups for trisomies 13, 18, and 21 (>1:150).

14 See *Bioethics* (2015) 29:1 (special issue on NIPT).

15 See Nuffield Council on Bioethics 2017; see: Ravitsky 2016.

16 Nuffield Council on Bioethics 2017, at Chapter 5.

17 Seminally, see: Johnston and Kaye 2004.

18 In *ABC v St George’s Healthcare NHS Trust & Ors* [2017] EWCA Civ 336, at para 47, Irwin LJ said that ‘it [should be] noted that neither party submits there is any previous case in this jurisdiction which has considered the obligation to disclose information arising from the practice of clinical genetics.’ To which, we might add, let alone the practice of *research* genetics. However, unlike the High Court, the Court of Appeal took the view that, even though the information at issue was subject to a duty of confidence, and even though there were ‘floodgates’ concerns (see paras 42–44), the claimant at least had an arguable case that it would be fair, just and reasonable to require disclosure in the circumstances.

19 [2015] UKSC 11.

20 For questions concerning this version of the story, see (Montgomery and Montgomery 2016).

21 See *Bolam v Friern Hospital Management Committee* [1957] 1 WLR 852, qualified by *Bolitho v City and Hackney Health Authority* [1998] AC 232.

22 [2015] UKSC 11, para 81.

23 [2015] UKSC 11, para 91.

24 Nb Lady Hale in *Montgomery* [2015] UKSC 11, at para 115.

25 Seminally, see: Llewellyn 1960; Adams and Brownsword 2006.

26 Compare Brownsword 2007.

27 Compare the prognosis in Greely 2016.

28 [2017] SGCA 38.

29 Albeit using a distinctive 3 stage approach ([2017] SGCA 38, para 132–134). At [2017] SGCA 38, paras 138–139, the Court states that the class of potentially material information is not ‘limited to risk-related information’. It also includes information relating to the diagnosis and prognosis, the nature of the proposed treatment, and the alternatives.

30 See [2017] SGCA 38, para 114, where the Court cites Beauchamp and Childress 2001, at 12.

31 [2017] SGCA 38, at para 124.

32 [2017] SGCA 38, at para 125.

33 [2017] SGCA 38, at para 135.

34 [2017] SGCA 38, at paras 150–152.

35 [2017] SGCA 38, at para 153.

36 [2017] SGCA 38, at para 150.

37 See, generally, Beyleveld and Brownsword 2007.

38 Compare: Dworkin 1978.

39 See, too, Article 5(c) of the UNESCO Declaration on the Human Genome (endorsing the ‘right of every individual to decide whether or not to be informed of the results of genetic examination and the resulting consequences’).

40 See: http://www.geenivaramu.ee/for-donors/gene-donor-consent-form.html (last accessed December 1, 2013).

41 See: e.g. the survey in Ethics and Governance Council of UK Biobank 2015.

42 For evidence of such an expectation, see, e.g. Bovenberg et al. 2009 and Wellcome Trust and Medical Research Council 2012.

43 But, n.b., our general caveat about the need for sensitivity in relation to global variation in these normative concepts and culturally-bound values and priorities.

44 Compare: Beyleveld and Brownsword 2015, at 173 (for an argument based on agential self-understanding).

45 Compare Halliburton 2009; and J. Husted, ‘Autonomy and a Right Not to Know’, in Chadwick et al. 2014, at 24.

46 Ibid.

47 Clearly, this line of argument intersects with debates about proprietary rights in detached body parts (see: e.g. Brownsword 2009) and in personal data (see: e.g. Lanier 2013).

48 Compare Graeme Laurie, ‘Privacy and the Right Not to Know: A Plea for Conceptual Clarity’, in Chadwick et al. 2014, at 38.

49 [2015] EWHC 1394 (QB), [2017] EWCA Civ 336 (n 1 above).

50 See: Hall et al. 2009.

51 Nuffield Council on Bioethics 2017, at para 6.16.

52 See Deans et al. 2015.

53 See, e.g. Lessig 2006.

54 Nuffield Council on Bioethics 2017, at para 6.13. But, nb, Huntington’s Disease is a late onset condition and disclosure of this condition is precisely the question in the *ABC* case (n 1 above).

55 Nuffield Council on Bioethics 2017, at para 6.12. Again, the particular characteristics of the environment will reflect local conditions: see Mozersky et al. 2017.

56 See: Nuffield Council on Bioethics 1993, at para 5.8, where we read:
We begin by examining the issue of confidentiality, considering in particular why it is important and how it is currently protected by the law and by other means. We then ask whether there are circumstances in which the confidentiality of genetic information ought properly to be overridden to permit disclosure to those interested third parties who, it is sometimes said, have a ‘right to know.’
Although the Council clearly would prefer interested third parties (usually in families) to be informed, the subsequent discussion of the ethical issues (in paras 5.24–5.28 of the 1993 report) displays a reluctance fully to embrace a claim right to know.

57 In *ABC v St George’s Healthcare NHS Trust & Ors* [2017] EWCA Civ 336, at paras 57–60, there is an extended and helpful reference to the decision of the New Jersey Superior Court in *Safer v Pack* (291 N.J. Sup. 619, 677 A. 2d 1188) 1996, where it was held that a physician did have a duty to warn the child of a patient of a genetic risk. Arguably, this assists the foetus-that-becomes-a-person who claims a right to know the results of an NIPT carried out on his or her mother.

58 Nuffield Council on Bioethics 2017, at para 6.6. Only for conditions/impairments that are actually being tested for.

59 Nb, too, *ABC v St George’s Healthcare NHS Trust & Ors* [2017] EWCA Civ 336, at para 38, where Irwin LJ suggests that, where there is no cure for a condition, an argument for disclosure based on autonomy would be of ‘reduced value’ because the ‘claimant would have no meaningful choices open to her.’

60 Compare Wolf et al. 2012.

61 Wallach 2015, at 10.

62 Ibid.
